# A daily end‐to‐end quality assurance workflow for MR‐guided online adaptive radiation therapy on MR‐Linac

**DOI:** 10.1002/acm2.12786

**Published:** 2019-12-04

**Authors:** Xinfeng Chen, Ergun Ahunbay, Eric S. Paulson, Guangpei Chen, X. Allen Li

**Affiliations:** ^1^ Department of Radiation Oncology Medical College of Wisconsin Milwaukee WI 53226 USA

**Keywords:** adaptive RT, daily end‐to‐end QA workflow, MR‐Linac

## Abstract

**Purpose:**

Magnetic Resonance (MR)‐guided online adaptive radiation therapy (MRgOART), enabled with MR‐Linac, has potential to revolutionize radiation therapy. MRgOART is a complex process. This work is to introduce a comprehensive end‐to‐end quality assurance (QA) workflow in routine clinic for MRgOART with a high‐magnetic‐field MR‐Linac.

**Materials and Method:**

The major components in MRgOART with a high‐magnetic field MR‐Linac (Unity, Elekta) include: (1) a patient record and verification (R&V) system (e.g., Mosaiq, Elekta), (2) a treatment session manager, (3) an offline treatment planning system (TPS), (4) an online adaptive TPS, (5) a 1.5T MRI scanner, (6) an 7MV Linac, (7) an MV imaging controller (MVIC), and (8) ArtQA: software for plan data consistency checking and secondary dose calculation. Our end‐to‐end QA workflow was designed to test the performance and connectivity of all these components by transferring, adapting and delivering a specifically designed five‐beam plan on a phantom. Beams 1–4 were designed to check Multi‐Leaves Collimator (MLC) position shift based on rigid image registration in TPS, while beam 5 was used to check daily radiation output based on image pixel factor of MV image of the field. The workflow is initiated in the R&V system and followed by acquiring and registering daily MRI of the phantom, checking isocenter shift, performing online adaptive replanning, checking plan integrity and secondary 3D dose calculation, delivering the plan while acquiring MV imaging using MVIC, acquiring real‐time images of the phantom, and checking the delivering parameters with ArtQA**.**

**Results:**

It takes 10 min to finish the entire end‐to‐end QA workflow. The workflow has detected communication problems, permitted resolution prior to setting up patients for MRgOART. Up to 0.9 mm discrepancies in isocenter shift based on the image registration were detected. ArtQA performed the secondary 3D dose calculation, verified the plan integrity as well as the MR‐MV isocenter alignment values in TPS. The MLC shapes of beam 1–4 in all adaptive plans were conformal to the target and agreed with MV images. The variation of daily output was within ±2.0%.

**Conclusions:**

The comprehensive end‐to‐end QA workflow can efficiently check the performance and communication between different components in MRgOART and has been successfully implemented for daily clinical practice.

## 1 INTRODUCTION

Magnetic Resonance (MR)‐guided online adaptive radiation therapy (MRgOART) with MR‐Linac 1, 2, 3 is now clinically available. This technology has potential to significantly improve radiation therapy (RT) outcomes by providing excellent soft‐tissue contrast images, biological and functional information, and high‐quality real‐time image guidance during RT delivery. Compared to conventional RT, MRgOART with MR‐Linac is a more complex process involving not only the treatment delivery system and the patient R&V system, but also online MR imaging system, online treatment planning system (TPS) and online quality assurance (QA) system. The clinical MR‐Linac workflow relies on communication between systems on both the local machine and hospital networks. Any communication failures or interruption could impede patient treatment, significantly increase patient‐on‐table time, or result in abortion of the patient treatment, all of which, would potentially circumvent the advantages of MRgOART. Therefore, It is critical to check all the functionalities and communications of each major component of the MR‐Linac system in advance to ensure accurate and efficient MRgOART in the clinic.

In this report, a daily End‐to‐End QA workflow was designed to provide a fast approach to test functionality and connectivity of all components of MRgOART on MR‐Linac. This includes tests on acquiring and transferring of the daily MR images, image registration, online adaptive planning, online adaptive plan QA, adaptive plan delivery and motion monitoring during the delivery. In addition, the daily dosimetry output of the Linac is also checked in the workflow.

## 2 MATERIALS AND METHODS

All daily End‐to‐End QA workflow tests were carried out on a high‐field MR‐Linac system (Unity, Elekta) installed in our institution. The Unity system consists of a linear accelerator with a nominal 7 MV flattening filter‐free photon beam (160‐leaf Multi‐Leaves Collimator (MLC) oriented in fixed superior‐inferior direction), and a Philips 1.5 T integrated wide‐bore MRI scanner. The main magnetic field of the scanner is oriented transverse to the irradiation field. An inverse treatment planning system (TPS) (Monaco, v5.40.00, Elekta AB) is used for the MR‐Linac, employing a graphics processing unit (GPU)‐based Monte Carlo dose calculation algorithm 4 which considers dosimetric effects of the magnetic field.^5^

### 2.A Major components of the daily end‐to‐end QA workflow

The major components in the MRgOART daily QA workflow with the Unity system include: (1) a daily QA patient, (2) a patient record and verification (R&V) system, (3) a treatment session manager (TSM), (4) an offline TPS, (5) an online adaptive TPS, (6) a 1.5T MRI scanner, (7) a 7MV Linac, (8) an MV imaging controller (MVIC), and (9) ArtQA software.^6^

An MR‐MV alignment phantom with seven ZrO_2_ spheres surrounded by plastic and copper sulphate solution was used as the daily QA patient in the workflow. A 3D reference plan with five static‐field beams was generated with offline Monaco TPS as follows: a) The reference image was shifted by 1.0 cm, 1.2 cm and −1.5 cm in x, y and z directions, respectively; b) beams 1‐4: the MLC shapes were conformed to four ZrO_2_ spheres at different orthogonal gantry angles 0^0^, 90^0^, 180^0^ and 270^0^; c) beam 5: a 5 × 5 cm^2^ field with 100 MU at gantry 0^0^ was used for daily output check based on the fact that the image pixel factor (IPF) of the MV image is correlated to the accumulated radiation during MV imaging.

The patient R&V system used in our clinic is Mosaiq (Elekta, Stockholm). The pretreatment preparation was done through Mosaiq which includes plan import, plan of care configuration, creating treatment calendar, and scheduling treatment appointments.

In the Unity system, the couch cannot be shifted once the patient is positioned within the bore. Therefore, every fraction requires adaptive plan. There are two adaptive plan workflows^7^ available for offline and online TPS: i) adapt‐to‐position (ATP) in which the adaptive plan is generated based on the isocenter shift determined from rigid registration of daily and reference images, with the plan optimized on reference images, and ii) adapt‐to‐shape (ATS) in which the adaptive plan is generated based on daily anatomy and the plan is optimized on the daily image. Four strategies are provided for both ATP and ATS workflows, 1) original segments, 2) adapt segments (segment aperture morphing), 3) optimize weights and 4) optimize weights and shapes. The ATP workflow with adapt‐segments strategy was selected in the daily QA workflow.

The Elekta treatment session manager (TSM) for Unity includes TSM‐setup and TSM‐imaging workspaces. The TSM‐setup workspace is used for patient verification and setup confirmation. The TSM‐image workspace is used for daily imaging activities which includes pre‐treatment and post‐treatment imaging, verification imaging and motion monitoring etc.

The MVIC is the MV imaging system used to verify MLC shape and daily output in the workflow. The MV imaging panel consists of Gadox scintillator and 1024 × 1024 photo diodes. The imaging resolution is 1024 × 1024 pixels with the pixel size 0.21 mm at the isocenter plane. The IPF is inversely correlated with radiation during the MV imaging, thus can be used to measure Linac output.

*Ar*tQA is a software QA tool developed to check plan data integrity and dosimetry quality in a TPS, verify the plan data transfer from the TPS to patient R&V system, independently check the MU from TPS with secondary 3D dose calculation and verify the treatment delivery after completion. The category of checking includes patient demographics, study set, structures, beams, dose calculation and dose quality. For example, if the beam parameters (MU, gantry angle, MLC setting etc.) are changed in the patient R&V system by accident, they will be flagged in red when compared to TPS. The secondary 3D dose calculation is based on a modified Clarkson integration method and partially takes into account the dosimetric effect of magnetic field by using the commissioning beam data of the Unity system.^8^

### 2.B Major steps of the daily end‐to‐end QA workflow

The major steps of the daily QA workflow include the following:

1. In Mosaiq, the daily QA patient is selected, and the MR daily assessment is completed.
2. The TSM will automatically launch both TSM‐setup and TSM‐imaging windows upon acknowledging all the readiness checks in Mosaiq.
3. Once the QA patient is setup on the couch in the treatment room, the patient is verified, and the setup is confirmed in TSM‐setup window.
4. The daily pretreatment imaging is started from TSM‐imaging window.
5. Once the daily MR imaging is done and the images are transferred to online Monaco, the reference planning images of the QA patient will be opened in online Monaco, the daily images will be automatically loaded and rigidly registered with reference planning images.
6. The isocenter shift due to the registration in TPS is recorded and compared to the expected values. The difference reflects the combination of phantom setup error and the accuracy of the rigid registration.
7. The ATP workflow with the adapt‐segments strategy is used in the online TPS for the adaptive replanning.
8. The adaptive plan is checked with ArtQA once the replanning is finished, and the consistency of beam parameters is checked after the adaptive plan is approved and transferred into Mosaiq.
9. Motion monitoring (i.e., cine MR image acquisition) is started from the TSM‐imaging workspace.
10. The daily plan is delivered from Linac console, and the MVIC records the MV portal images of the phantom for different beams.
11. The MLC position is checked for each beam by comparing the MV images with the MLC shape in TPS.
12. The IPF of the MV image for beam of 5x5 field is obtained from MVIC and compared to the tolerance value to make sure that the daily MR‐Linac output is within the +/‐3% difference of the calibration.

### 2.C IPF response curve for daily output QA

The IPF response curve for daily output QA is generated and checked with Linac monthly and daily output QA setups. The output of the MR‐Linac in our clinic is calibrated as 100 cGy per 100 MU at the depth of the maximum dose (D_max_) (at 143.5 cm SAD, gantry = 0^0^) and the daily output may vary. The monthly output setup is used to verify the output, and the daily output QA setup is used to collect IPFs for a range of MUs to determine the response curve. The reference IPF value (corresponding to 100 cGy) and the tolerance values (corresponding to 100 ± 3 cGy) are extracted and compared with currently used values. The daily output QA sheet will be updated if the reference IPF value change is more than 0.5%.

The procedure to generate of the response for daily output QA is as follows: 1) the output at the moment of measurement is identified with monthly output QA setup consisting of virtual water and ion chamber surrounded by liquid water; 2) daily output QA setup with 5 × 5 cm^2^ field size and 97‐103 MU are delivered to the MR‐MV alignment phantom and the corresponding IPF values are recorded; 3) the MUs are converted into doses at D_max_; and 4) the relation between the IPF value and the dose is then extracted using linear least‐squares fit and the IPF values for 100, 97 and 103 cGy are recorded as the reference, the lower and upper tolerances of the output.

## 3 RESULTS

The daily end‐to‐end QA workflow has been used in our clinic since January 2019. The entire workflow can be completed within 10 min. The workflow has detected numerous communication failures, for example, the TSM could not be launched automatically when the workflow was initiated from patient R&V system; TSM could not communicate with the Philips MRI scanner to start the daily images; the daily images could not be imported to the online TPS for adaptive planning; motion monitoring images failed to initialize; the adaptive plan could not be delivered on the Linac. Detections of these failures enabled us to resolve issues prior to setting up patients for daily treatment. The results of isocenter shift due to the image registration in TPS, as well as IPF value for daily output check, were recorded in the QA database. The MLC positioning, motion monitoring, MV image quality, system communications, adaptive plan QA and treatment delivery were also checked during the workflow.

### 3.A Image registration in TPS

**Figure 1:**
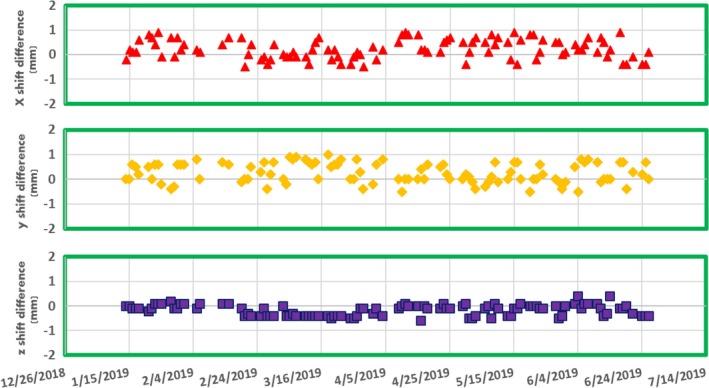
The differences of isocenter‐shift along X, Y, and Z axis between the expected and from daily registration in TPS.

The accuracy of the image registration was checked by comparing the daily image isocenter shift with the expected isocenter shift due to the offset of the reference image sets. As shown in Fig. 1, the difference of isocenter shift between the expected and daily registration in x, y and z directions were all <1.0 mm.

### 3.B Motion monitoring

**Figure 2:**
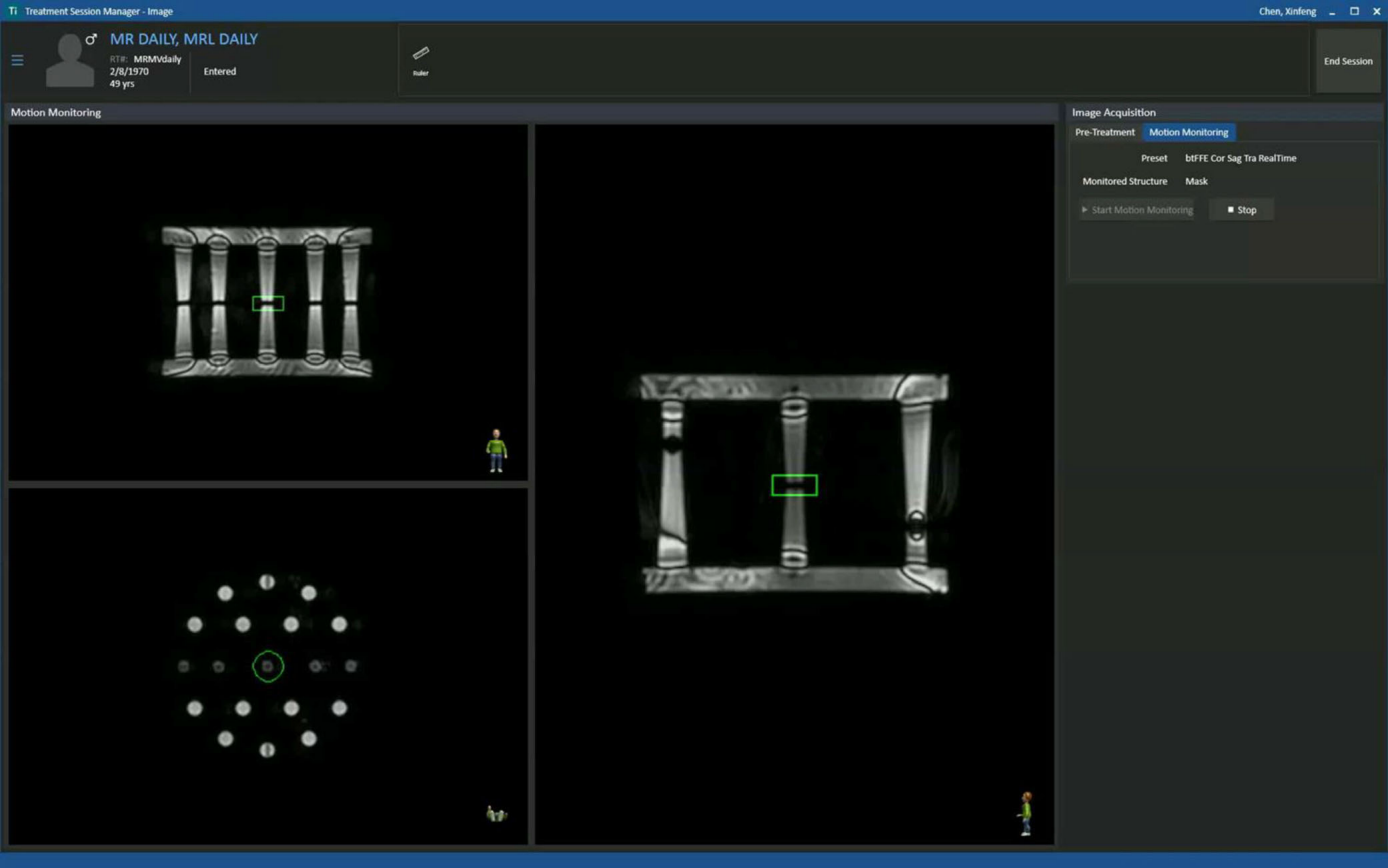
Tri‐planar 2D cine images acquired for motion monitoring in the TSM‐imaging window. (The green contour is the monitored structure).

Motion monitoring images were acquired with a tri‐planar 2D balanced turbo field echo cine sequence after the adaptive plan was generated and before it was delivered. Although the MR‐MV phantom is not a motion phantom, this test verifies the real‐time imaging functionality of the Unity through the TSM‐imaging workspace. As shown in Fig. 2, tri‐planar 2D cine images were acquired and displayed in real‐time in the TSM‐imaging window.

### 3.C Adaptive plan QA

**Figure 3:**
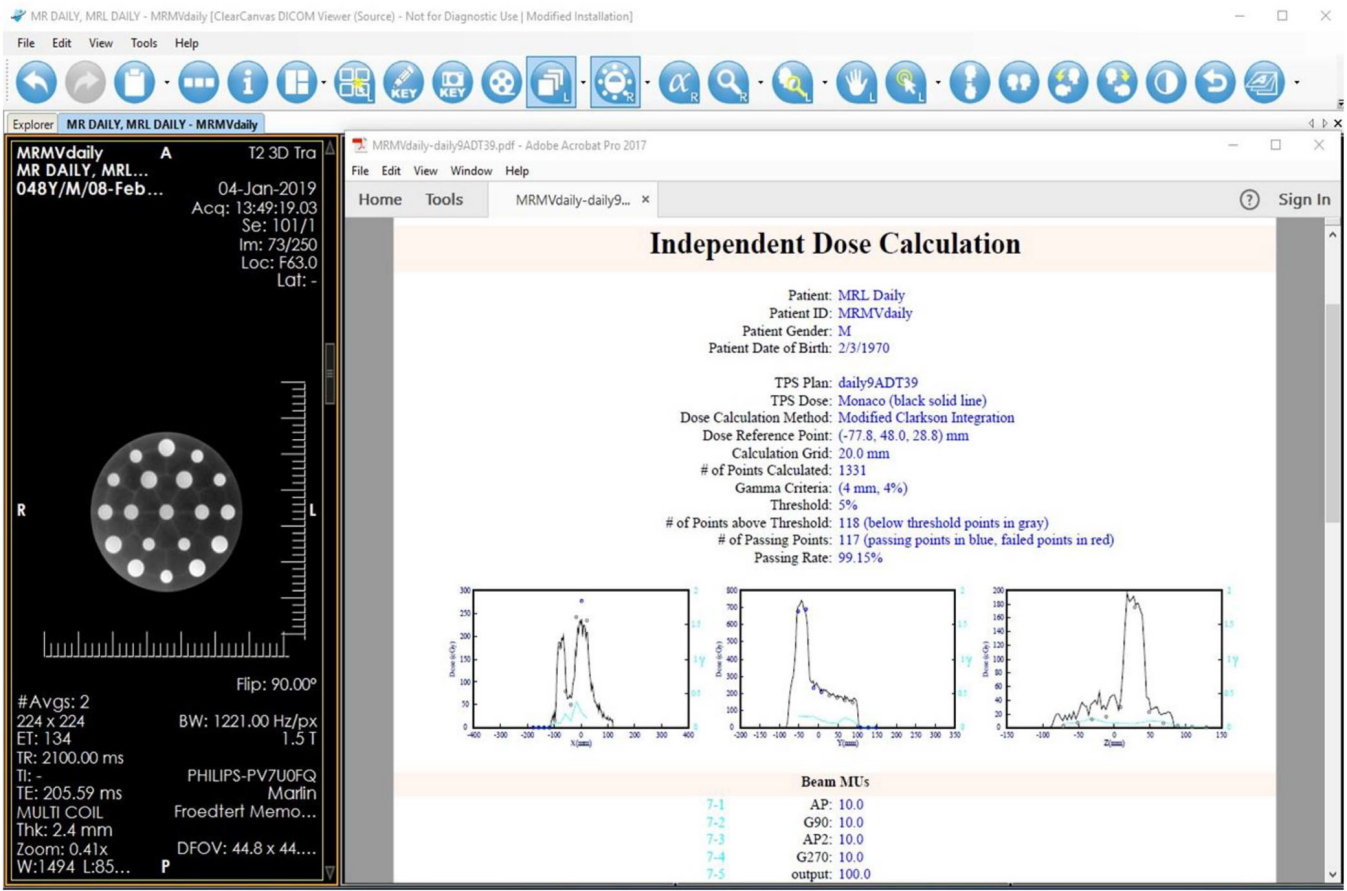
Example of independent secondary dose calculation with ArtQA.

After the adaptive plan was generated and saved, the second dose verification with *ArtQA* was performed and a summary of the dose calculation was automatically generated and saved in a pdf file as shown in Fig. 3. The summary included patient demographic information, dose calculation details, QA criteria and results, dose comparison plots along x, y, z axis with dose reference point as origin, and the beam MUs. Dose was compared using 3D gamma analysis with 4 mm distance to agreement and 4 % dose difference. The passing rates for all daily adaptive plans to date exceeded 99%.

**Figure 4:**
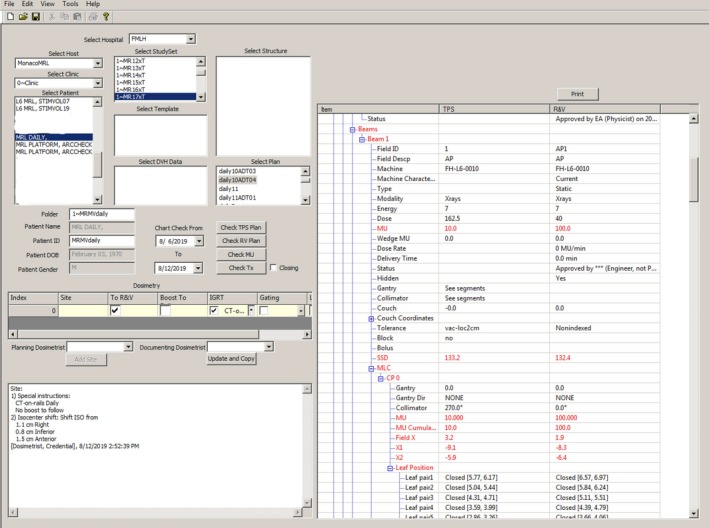
An example of the ArtQA check after the plan was transferred to Mosaiq (The inconsistent parameters have been flagged in red).

With ArtQA, the beam parameters in TPS were verified before the plan approval and the transferring consistency of the beam parameters from TPS to Mosaiq was checked after the approval. As shown in Fig. 4, the beam parameters including field, beam modality, energy, MU, gantry angle, MLC position and couch position were compared between the TPS and Mosaiq, with those inconsistent parameters flagged in red. The MR‐MV isocenter offset values were verified with ArtQA.

### 3.D MLC positioning

**Figure 5:**
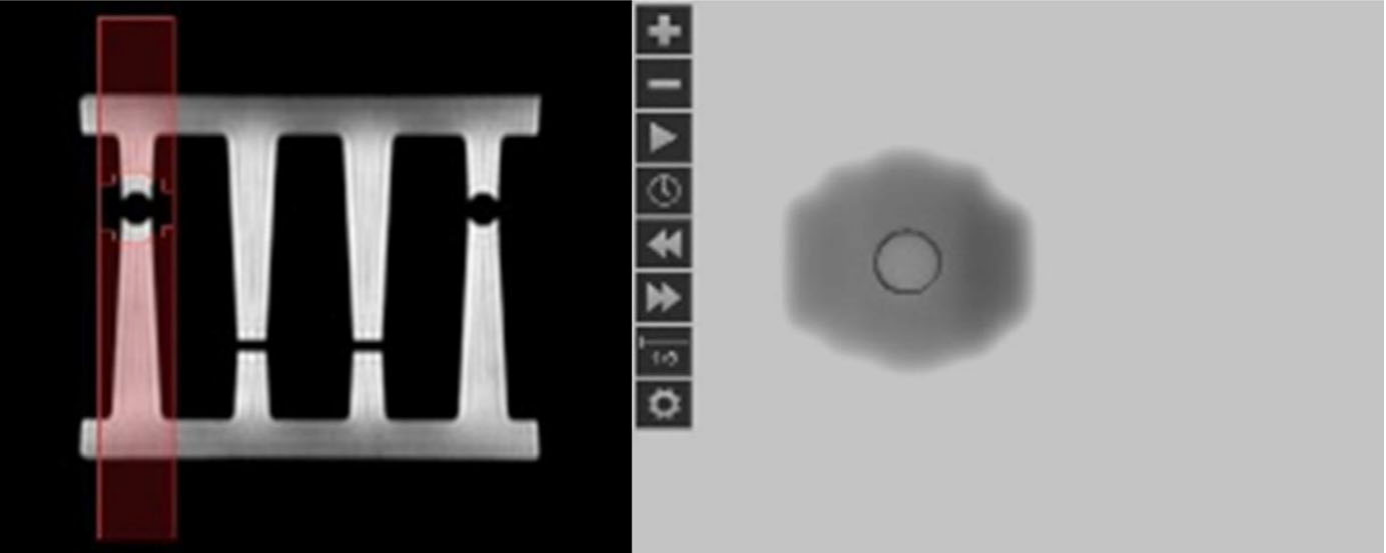
The comparison of the MLC shape for one of the beams in the workflow. (Left panel: The MLC shape with the target in TPS; Right panel: The corresponding portal MV image.). TPS, treatment planning system.

The MLC positioning was visually inspected by comparing respectively the MLC shapes for beams 1–4 in TPS with those on portal images obtained with MVIC. This test verifies the segment aperture morphing (SAM) algorithm 9 in the adaptive plan correctly moves MLC leaves based on registration of the daily and reference images. As shown in Fig. 5, the MLC shape for one of the beams from TPS in left panel was consistent with that from the portal image in the right panel.

### 3.E Daily output

**Figure 6:**
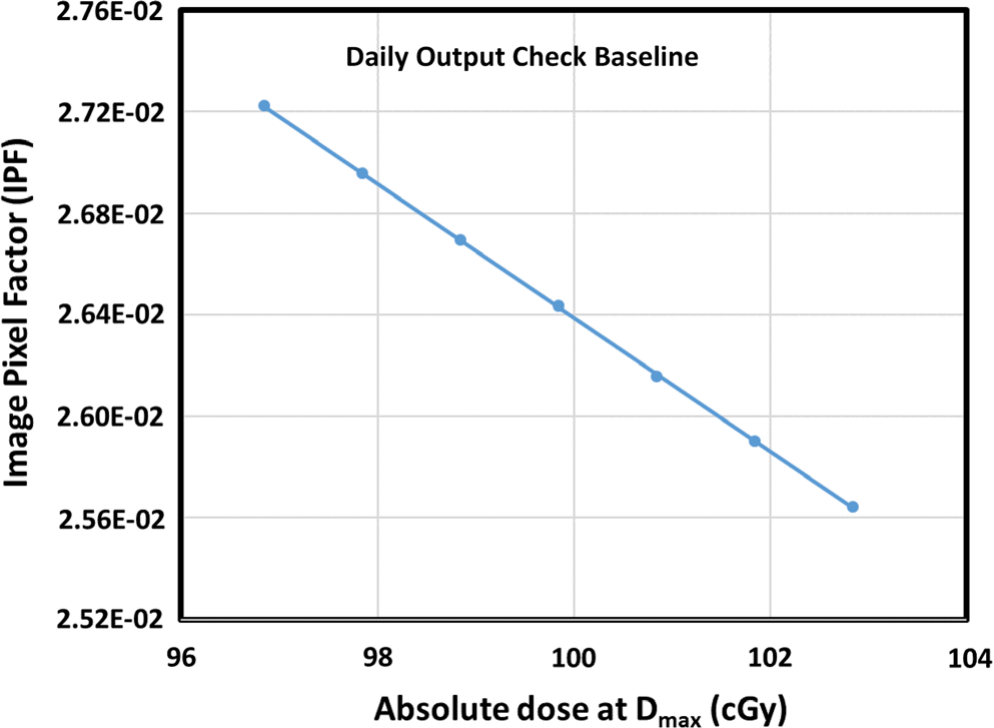
The IPF response curve which links the IPF with absolute output.

**Figure 7:**
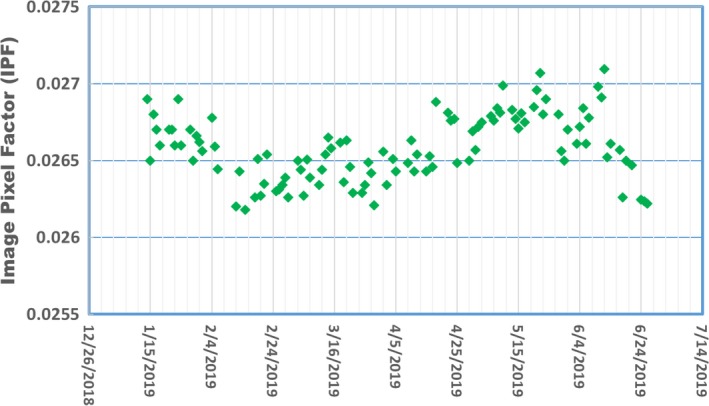
The daily IPFs obtained from MV portal images during the daily end‐to‐end QA workflow.

**Figure 8:**
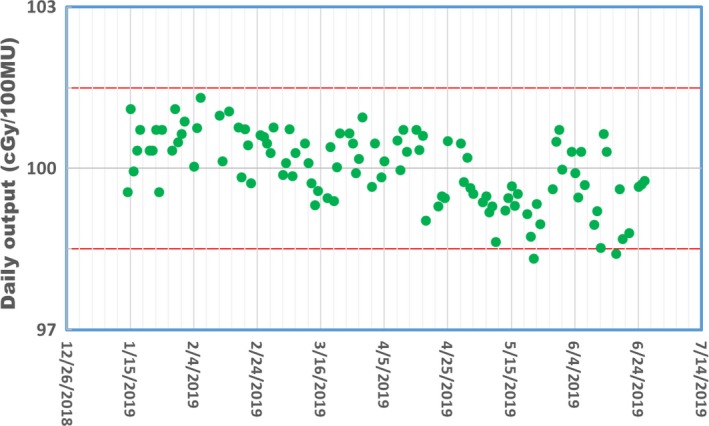
The daily outputs converted from IPFs obtained from daily end‐to‐end QA workflow.

The daily IPF was measured and converted into daily output using the IPF response curve as shown in Fig. 6. A summary of the daily IPF factors since January 2019 is shown in Fig. 7, and the corresponding daily output converted from the IPF in the same period is shown in Fig. 8. The maximum variation of the daily output was <2%.

### 3.F IPF response

**Figure 9:**
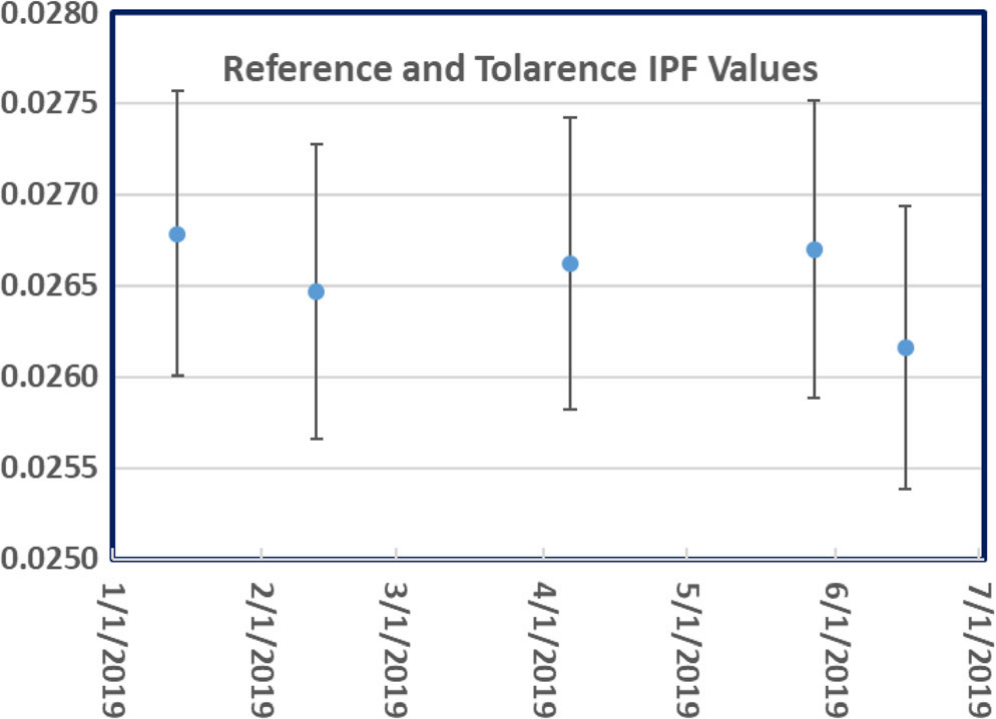
The IPF reference and tolerance values obtained at different time during Monthly QA.

The IPF reference and tolerance values extracted at different times are plotted in the Fig. 9. The change of the IPF reference value was less than 1.2% except for the last measurement on June 1, when a new magnetron was installed. The IPF value changed approximately 2% at that time, indicating the regeneration of the IPF response curve was required to account for the change of the major dosimetry components of the Linac.

## 4 SUMMARY

The communication between various components of Unity MR‐Linac system during MRgOART workflow (including the patient R&V system, treatment session manager (TSM), MR imaging console, online TPS and Linac console, image registration in TPS, adaptive planning and QA, motion monitoring, MLC conformality, and radiation output) can be checked in 10 min with the newly developed daily end‐to‐end QA workflow. The accuracy of the image registration is obtained by comparing the daily image isocenter shift from the reference images to expected values. The differences in x, y and z directions should be less than 1 mm. The second 3D dose calculation for adaptive plan is carried out with *ArtQA* and is compared with TPS calculation using absolute gamma criteria. The consistency of the plan parameter transferring from TPS to patient R&V system, and to Linac console is also verified in the workflow.

The daily output is extracted with image pixel factor (IPF) of the MV portal image. The variation of the daily output was found to be within +/− 2% of the nominal values. Since the response of the MVIC detectors may change with time, it is necessary to verify the IPF response monthly, and update the daily output QA criteria if the change of the reference IPF value is more than 0.5%.

In summary, a daily end‐to‐end QA workflow has been successfully implemented in our clinic since January 2019. The entire QA workflow can be performed with existing Unity system software and hardware except for the ArtQA, which was developed in‐house. The workflow verifies the performance and communication between different Unity subsystems in a short time frame, ensuring accurate and efficient execution of MRgOART in the clinic.

## CONFLICT OF INTEREST

The authors do not have any conflict of interest to report.
